# Non-classical monocyte levels correlate negatively with HIV-associated cerebral small vessel disease and cognitive performance

**DOI:** 10.3389/fcimb.2024.1405431

**Published:** 2024-10-23

**Authors:** Meera V. Singh, Md Nasir Uddin, Mae Covacevich Vidalle, Karli R. Sutton, Zachary D. Boodoo, Angelique N. Peterson, Alicia Tyrell, Madalina E. Tivarus, Henry Z. Wang, Bogachan Sahin, Jianhui Zhong, Miriam T. Weber, Lu Wang, Xing Qiu, Sanjay B. Maggirwar, Giovanni Schifitto

**Affiliations:** ^1^ Department of Neurology, University of Rochester, Rochester, NY, United States; ^2^ Department of Microbiology and Immunology, University of Rochester, Rochester, NY, United States; ^3^ Department of Biomedical Engineering, University of Rochester, Rochester, NY, United States; ^4^ Clinical and Translational Science Institute, University of Rochester, Rochester, NY, United States; ^5^ Department of Imaging Sciences, University of Rochester, Rochester, NY, United States; ^6^ Department of Neuroscience, University of Rochester, Rochester, NY, United States; ^7^ Department of Physics and Astronomy, University of Rochester, Rochester, NY, United States; ^8^ Department of Obstetrics and Gynecology, University of Rochester, Rochester, NY, United States; ^9^ Department of Biostatistics and Computational Biology, University of Rochester, Rochester, NY, United States; ^10^ Department of Microbiology, Immunology and Tropical Medicine, George Washington University, Washington, DC, United States; ^11^ Department of Electrical and Computer Engineering, University of Rochester, Rochester, NY, United States

**Keywords:** non-classical monocytes, cerebral small vessel disease, HIV/AIDS, cognitive impairment, MRI

## Abstract

**Background:**

Despite antiretroviral treatment (cART), aging people living with HIV (PWH) are more susceptible to neurocognitive impairment (NCI) probably due to synergistic/additive contribution of traditional cerebrovascular risk factors. Specifically, transmigration of inflammatory CD16+ monocytes through the altered blood brain barrier (BBB) may exacerbate cerebral small vessel disease (CSVD), a known cause of vascular cognitive impairment.

**Methods:**

PWH on cART (n=108) and age, sex, and Reynold’s cardiovascular risk score-matched uninfected individuals (PWoH, n=111) were enrolled. This is a longitudinal observational study but only cross-sectional data from entry visit are reported. Neuropsychological testing and brain magnetic resonance imaging (MRI) were performed. CSVD was diagnosed by Fazekas score ≥1. Flow cytometric analyses of fresh whole blood were conducted to evaluate circulating levels of monocyte subsets (classical, intermediate, and non-classical) and markers of monocyte activation (CCR2, CD40, PSGL-1, TNFR2 and tissue factor). ELISAs were used to measure sCD14, ICAM, and Osteoprotegerin. Two-way analysis of variance (ANOVA), and linear regression models were performed to study the effects of HIV status, CSVD status, and their interaction to outcome variables such as cognitive score. Two-sample t-tests and correlation analyses were performed between and within PWoH with CSVD and PWH with CSVD participants.

**Results:**

PWH with CSVD (n=81) had significantly lower total cognitive scores, higher levels of NCMs and soluble CD14 and intracellular adhesion molecule 1 (ICAM-1) as compared to PWoH with CSVD group (n=68). sCD14 and ICAM1 were positively correlated with each other indicating that monocyte and endothelial activation are associated with each other. Cognition was negatively correlated with NCMs, especially in the PWH with CSVD group. Among other blood biomarkers measured, osteoprotegerin levels showed mild negative correlation with cognitive performance in individuals with CSVD irrespective of HIV status.

**Conclusions:**

Elevated levels of NCMs may contribute to neuroinflammation, CSVD and subsequent cognitive impairment. This finding is of particular relevance in aging PWH as both HIV and aging are associated with increased levels of NCMs. NCMs may serve as a potential biomarker to address these comorbidities. Further longitudinal studies are needed to evaluate whether changes in NCM levels are associated with changes in CSVD burden and cognitive impairment.

## Introduction

HIV-associated age-related comorbidities have a taken a center-stage in the management of this disease, owing to the increased longevity of people living with HIV (PWH) in the post-combination anti-retroviral therapy (cART) era. Among these, cerebral small vessel disease (CSVD), a common condition associated with aging, is known to be more prevalent in PWH than in the general population, as reported by our group (Murray et al., 2021) and others (Moulignier et al., 2018). CSVD affects small penetrating arteries, arterioles, capillaries, and small veins. It covers a range of pathological and clinical abnormalities such as small subcortical infarcts, lacunes, white matter hyperintensities (WMH), enlarged perivascular spaces (EPVS), cerebral microbleeds (CMB) and brain atrophy that can only be detected *in vivo* by neuroimaging techniques (Pantoni, 2010; Wardlaw et al., 2013) or histologically in postmortem tissue. CSVD is considered to be a major contributor to vascular cognitive impairment (VCI) in the older population (Moulignier et al., 2018; Li et al., 2021).

Inflammation can alter the blood-brain barrier (BBB) permeability and the overall function of the neurovascular unit. Thus, inflammation is a recognized contributor to CSVD (Low et al., 2019; Wardlaw et al., 2019). In addition to markers of systemic inflammation, a relatively small study has found an association between pro-inflammatory monocytes and CSVD progression (Noz et al., 2021). Several reports have also shown either higher levels of circulating monocytes or soluble monocyte products to be elevated during cognitive decline associated with sepsis, Alzheimer’s disease, and stress (Andonegui et al., 2018; Berger et al., 2019; Kuan et al., 2021; Munawara et al., 2021). In the context of HIV infection, monocytes are of interest as they can mediate both coagulopathy and neuroinflammation (Burdo et al., 2010; Valcour et al., 2012; Schechter et al., 2017). Therefore, it is plausible that aging and HIV may have an additive or synergistic effect on CSVD (Haddow et al., 2014; Morgello et al., 2014; Soontornniyomkij et al., 2014), which is in part sustained by pro-inflammatory monocytes.

There are three subsets of circulating monocytes defined by the difference in expression pattern for CD14 and CD16 as classical (CD14+CD16-), intermediate (CD14+CD16+) (IMCs), and non-classical (CD14loCD16++) monocytes (NCMs). It has been suggested that monocytes sequentially mature from classical to intermediate to non-classical phenotype during circulation (Patel et al., 2017; Tak et al., 2017). We hypothesized that CD16+ monocytes (IMCs and NCMs) were more likely to be involved in the pathogenesis of CSVD and CSVD associated cognitive impairment. Given the role played by the BBB in CSVD, we also hypothesized that markers of endothelial injury would correlate with markers of monocyte activation and cognitive performance. Here, we report the cross-sectional data in a study that enrolled PWH, and age and sex matched people without HIV (PWoH) in the context of CSVD.

## Materials and methods

### Standard protocol approvals, registrations, and patient consents

The study was approved by the University of Rochester Research Subjects Review Board. Written informed consent was obtained from all study participants before enrollment into the study as per the Declaration of Helsinki.

### Study design

This is a longitudinal observational study. Only cross-sectional data from the entry study visit are reported here. The longitudinal data analysis is in progress and has not been included in this report. Inclusion and exclusion criteria and all the study procedures as well as sample size justification are described in detail in our previous report (Murray et al., 2020). Briefly, inclusion criteria for PWH included, stable cART for at least 6 months prior to screening and age ≥ 18. We excluded individuals with symptomatic cardiovascular disease (CVD) such as angina, myocardial infarction, stroke or other peripheral atherosclerotic disease and uncontrolled vascular risk factors, such as diabetes mellitus and hypertension. We also excluded those with severe premorbid or comorbid psychiatric disorders, schizophrenia, bipolar disorder, and active depression. Brain infections other than HIV-1 and space-occupying brain lesions were also exclusionary. Individuals with dementia from any cause were not enrolled. For safety reasons, those with metallic implants were excluded. The PWoH population differed from PWH only in HIV status. A total of 108, PWH on cART and 111, age, sex, and Reynold’s cardiovascular risk score-matched uninfected individuals (PWoH) were enrolled.

### Neuropsychological battery

All cognitive testing was performed by staff trained and supervised by a clinical neuropsychologist. We administered a comprehensive neuropsychological battery that included tests of Attention and Working Memory (CalCAP), Processing Speed (Symbol Digit Modalities Test and Stroop Color Naming), Executive Function (Trail making Test Parts B, Stroop Interference task), Fine Motor Skills (Grooved Pegboard, left and right hand); Verbal and visual learning (Rey Auditory Verbal Learning Test AVLT (Trials 1-5), Rey Complex Figure Test Immediate Recall), verbal and visual memory (Rey Auditory Verbal Learning Test RAVLT Delayed Recall, Rey Complex Figure Test Delayed Recall) and language (verbal fluency tests) at each visit. Premorbid intellectual functioning ability was estimated via WRAT-4 Reading at the baseline visit only.

Raw scores from each test were converted to z-scores using test manual norms. We created cognitive domain scores by averaging the z-scores of each test within each domain. A total cognitive score was calculated by summing the six cognitive domain z-scores (Attention and Working Memory, Processing Speed, Executive Function, Fine Motor Skill, Verbal and Visual Learning, Verbal and Visual Memory, and Language). We used Frascati criteria (Antinori et al., 2007) to determine HAND diagnoses for each participant.

### MRI acquisition, analysis, and CSVD diagnosis

Imaging was conducted on a Siemens 3T MAGNETOM PrismaFit whole-body MRI scanner (Erlangen, Germany; software version VE11c) equipped with a 64-channel phased array head coil. Detailed study protocols have already been reported by our group (Murray et al., 2020). Briefly, the following imaging was acquired: high-resolution 3D T1-weighted (T1w) anatomical images using a magnetization prepared rapid gradient echo (3D MPRAGE) sequence (inversion time -TI=926 ms; repetition time/echo time -TR/TE=1840/2.45 ms; image resolution = 1 × 1 × 1 mm^3^); 2D T2-weighted (T2w) images using a turbo spin echo sequence (TR/TE = 6000/100 ms; image resolution = 0.5 × 0.5 × 5 mm^3^); 3D fluid-attenuated inversion recovery (FLAIR) image (TI = 1800 ms; TR/TE = 5000/100 ms; resolution = 1 × 1 × 1 mm^3^); and 3D T2*-weighted images using a multi-echo gradient echo (MEGE) sequence with bi-polar readout (TR/TE 1st echo = 84/5.43 ms; number of echoes = 8; image resolution = 0.94 × 0.94 × 2.0 mm^3^). The T1w, T2w, T2*w and FLAIR images were used to evaluate CSVD burden by an experienced neuroradiologist using the Fazekas score (CSVD+ if Fazekas score ≥1) (Fazekas et al., 1987). Fazekas score is a qualitative neuroimaging scale used to assess the severity of WMH in the brain. Finally, VolBrain (Manjón and Coupé, 2016) was used to quantitatively measure the total WMH burden using the T1w and FLAIR images.

### Whole blood collection and processing

Approximately, 40 ml of whole blood was collected in Acid Citrate Dextrose (ACD) vacutainers, incubated at room temperature with slow shaking, and processed within 2 hours of collection. Plasma was isolated by centrifugation at 1000 X g for 10 minutes at room temperature. Plasma was aliquoted and cryopreserved at -80 °C and used to perform ELISA analyses mentioned below. 1ml of whole blood was processed by flow cytometry to measure different monocyte subsets and expression of monocyte activation markers such as c-c chemokine receptor 2 (CCR2), CD40, p-selectin glycoprotein ligand (PSGL-1), tumor necrosis factor receptor 2 (TNFR2) and tissue factor (TF) as described previously (Murray et al., 2020). Please note that we are missing data from whole blood flow cytometry analyses and ELISA analyses from 4 participants in PWoH group and 4 participants in PWH group due to instrument failure or inability to obtain blood samples.

### Flow cytometric analysis of whole blood

Within two hours of the blood draw, whole blood was fixed with paraformaldehyde (PFA) followed by RBC lysis using ACK lysis buffer. The cells were then washed and stained with titrated amounts of antibodies against anti-CD14 PE (BD Biosciences # 555398; 10 μl), anti-CD16 PE Cy7 (BD Biosciences 557744; 3 μl), anti-PSGL-1 FITC (R&D Systems # FAB9961G; 1.5 μl), anti-CD40 FITC BD Biosciences. # 555598; 10 μl), anti-CCR2 FITC (R&D Systems # FAB151G; 1.5 μl), anti-TF FITC (Miltenyi Biotech Inc # REA949, 10μl) and anti-TNFR2 FITC (Miltenyi Biotech #130-107-743; 1μl) for 30 minutes at room temperature in dark. The cells were washed and acquired using Accuri C6 flow cytometer. 75,000 gated leukocytes were acquired based on forward and side scatter per tube. Data was analyzed using Flow Jo software version 10.4.2. Florescence minus one (FMO) controls such as unstained cells, cells stained with anti-CD14 and anti-CD16 were used to gate on three subsets of monocytes, classical monocytes (CD14+ CD16-), intermediate monocytes (CD14+CD16+; IMMs) and non-classical monocytes (CD14lo CD16+, NCMs). Expression of CCR2, CD40, PSGL-1, TNFR2 and TF was measured on all monocyte subsets. In depth information about the gating strategy used for data analysis is described in detail in our previous report using the same cohort (Murray et al., 2020).

### ELISAs

Plasma samples were used to conduct ELISA assays for ICAM (# DY720-05, R and D systems, MN, USA), VCAM (# DY809, R and D systems, MN, USA), sCD14 (# DY383, R and D systems, MN, USA), CD163 (# DY1607, R and D systems, MN, USA), Osteoprotegerin (# DY805, R and D systems, MN, USA) and lipoprotein associated phospholipase A2 (LpPLA2) (# ab235643, Abcam, Cambridge, UK) as per manufacturer’s instructions.

### Statistical analysis

The descriptive characteristics of study participants are shown in **Table 1**. Percentages and frequencies for discrete variables as well as the means and standard deviations for continuous variables were reported. Two-way analysis of variance (ANOVA) was performed to study the effects of HIV infection, CSVD status, and their interaction, to the cognitive status of subjects. HIV status was included because it significantly impacts cognitive performance, as confirmed by our study (**Table 2**). CSVD status was included because CSVD is associated with brain structural changes that might lead to reduced cognitive performance. Multiple linear regression models were used to examine linear associations between a continuous outcome variable and several continuous and/or categorical covariates. For example, we investigated the association between NCMs (a continuous regressor) and total cognitive scores (a continuous outcome variable), controlling for HIV infection (binary), CSVD status (binary), and their interactions, and summarized the results in **Table 3**. The D’Agostino & Pearson test was performed to analyze the normality of the data. Based on the normality results, Two-sample Welch t-test (parametric) or Mann-Whitney U test (nonparametric) was used to perform two group comparisons (PWoH with CSVD group and PWH with CSVD group). Correlation analyses were done using Spearman’s rank correlation test. A p-value of less than 0.05 was considered significant. Error bars represent mean ± standard deviation. All statistical analyses were performed with GraphPad Prism 9.4.1 (GraphPad Software, Inc., San Diego, CA) and the R programming language 4.3.0 (R Foundation for Statistical Computing, Vienna, Austria).

**Table 1:** Demographic table.

Category	Variable	PWoH	PWH	P value
Demographic	N	108	111	
	Age	51 (17)	53 (11.271)	0.2835
	Sex (%F)	27.9	25.92	0.8736
	Hispanic	6.48	10.81	0.3106
	W: B: O	82.4:10.18:7.4	52.25:33.33:14.41	<0.0001
Clinical	Diabetes type II*	0.925	9.909	0.0097
	Hepatitis C*	0.925	9.909	0.0097
	Hypertension*	24.07	31.53	0.1577
	Reynold’s Risk Score	6 (6.519)	7 (6.299)	0.69
HIV	CD4+ count	–	662 (310)	–
	Viral Load	–	9 (18)	–
	cART	–		–
	INSTIs	–	90	–
	NNRTIs	–	15	–
	PI	–	20	–
CSVD	DWMH*	58.33	72.07	0.0379
	PVWMH*	20.37	35.1	0.0261
	WMH volume	0.1794 (0.3408)	0.4274 (0.7476)	0.0047
	N (Fazekas Score ≥1)	68	81	–
Total Cognitive Score		1.358 (4.782)	-1.810 (4.216)	<0.0001
HAND	No HAND	53.71	36.93	0.015
	ANI	42.59	49.54	–
	MND	3.7	12.61	–
	HAD	0	0.92	–

Where appropriate, values are presented as mean (standard deviation). Sex is presented as % Female; One PWoH study participant identified as transgender. Hispanic numbers are presented as % Hispanic. One PWoH and one PWH study participant did not report about Hispanic heritage. W: B: O corresponds to % White: % Black: % Other/Not Reported. Other categorical variables* are presented as %True. cART, combination antiretroviral therapy; INSTI, integrase strand transfer inhibitors; NNRTI, non-nucleoside reverse transcriptase inhibitors; PI, protease inhibitors. DWMH, deep white matter hyperintensity; PVWMH, periventricular white matter hyperintensity. Data presented as % of participants with DWMH or PVWMH burden. HIV Associated Neurocognitive Disorder (HAND): Asymptomatic Neurocognitive Impairment (ANI), Mild Neurocognitive Disorder (MND), HIV-associated Dementia (HAD). Units: Age (years); CD4+ Count (Count/mm^3^); Viral Load (Copies/mL); Absolute WMH Volume (cm^3^). Statistical analysis using GraphPad Prism V10 by Welch’s t test or fisher’s exact test.

**Table 2:** Two-way ANOVA to measure the effects of HIV status, CSVD status, and their interactions on total cognitive score.

Domain Name		Estimate	Std. Error	t value	P value	2.5%	97.5%
Total Z score	HIV Status	**-2.7664**	**1.1229**	**-2.4636**	**0.0146**	**-4.9804**	**-0.5525**
	CSVD Status	-0.6658	0.9181	-0.7252	0.4692	-2.4760	1.1444
	HIV-CSVD Interaction	-0.4587	1.3539	-0.3388	0.7351	-3.1280	2.2106

Values in bold denote statistical significance.

**Table 3:** Linear regression of total cognitive score as a function of NCM, Osteoprotegerin, ICAM levels, HIV status, and CSVD status.

	Total cognitive Z score					
	Estimate	Std. Error	t value	P value	2.5%	97.5%
% NCMs	-0.1085	0.0582	-1.8634	0.0642	-0.2235	0.0065
HIV Status	-2.9270	0.7450	-3.9287	0.0001	-4.3979	-1.4562
CSVD Status	-0.7531	0.7554	-0.9970	0.3202	-2.2444	0.7382
HIV-CSVD interaction	-0.6407	1.5111	-0.424	0.6721	-3.624	2.3427
Osteoprotegerin (log_10_)	-4.6882	1.9339	-2.4242	0.0162	-8.5021	-0.8743
HIV Status	-3.3468	0.6107	-5.4799	0.0000	-4.5513	-2.1423
CSVD Status	-0.9542	0.6626	-1.4402	0.1514	-2.2609	0.3525
HIV-CSVD interaction	0.5027	1.3372	0.3759	0.7074	-2.1347	3.14
ICAM (log_10_)	-2.1196	1.7212	-1.2315	0.2196	-5.5137	1.2745
HIV Status	-2.9542	0.6545	-4.5139	0.0000	-4.2448	-1.6636
CSVD Status	-1.0535	0.6845	-1.5390	0.1254	-2.4033	0.2963
HIV-CSVD interaction	-0.2731	1.3803	-0.1978	0.8434	-2.995	2.4488

### Data availability

Anonymized data not published within this article will be made available by request from any qualified investigator.

## Results

### Demographics, cognitive function, and CSVD burden of study population

As shown in **Table 1**, the PWoH and PWH cohorts were comparable for age and sex distribution as well as Reynold’s cardiovascular risk score (RRS). PWH had significantly lower cognitive performance and a higher percentage met criteria for mild neurocognitive disorder (MND) than PWoH. PWH were more likely to have diabetes or hepatitis C compared to PWoH (10% vs. 1%). 81 of the 111 PWH and 68 of the 108 PWoH had CSVD (as defined by Fazekas score ≥ 1). **Supplementary Figure 1A** shows the age distribution of the study participants when they were divided based on HIV and CSVD status. CSVD- groups (PWoH 44.08 years ± 15.31 years and PWH 48.13 years ± 13.09 years) were younger compared to the CSVD+ groups (PWoH 54.75 years ± 16.17 years and PWH 54.62 years ± 10.05 years). Similar distributions were seen for RRS (**Supplementary Figure 1B**). RRS in CSVD- PWoH was 4.025 ± 4.44 and in PWH without CSVD was 4.233 ± 3.99. The RRS was higher in those with CSVD. In PWoH with CSVD it was 7.831 ± 7.173 and in PWH with CSVD it was 7.654 ± 6.751. These differences in the distribution of CSVD and RRS reflect their known association with age.

In addition to the Fazekas score, WMH lesion volume was quantified via VolBrain. The PWH group had higher WMH lesion volume (0.4274 cm^3^± 0.7476 cm3) as compared to the PWoH group (0.1794 cm^3^ ± 0.3408 cm^3^) Data is shown in **Supplementary Figure 1C** (p=0.0047, 95% CI for difference between means: 0.0772 to 0.4187). Even among individuals that had CSVD (as assessed by Fazekas score), PWH with CSVD group showed higher WMH lesion volume (0.4989 cm^3^ ± 0.8014 cm^3^) as compared to PWoH without CSVD group (0.2412 cm^3^ ± 0.3888 cm^3^). Data is shown in **Supplementary Figure 1D** (p=0.0156, 95% CI for difference between means: 0.0496 to 0.4657). It should be noted that values from individuals that had no WMH lesion (0 cm^3^) were not included in the analysis (n=26 PWH and n=16 PWoH had 0 values and were excluded). Further, data from four PWoH study participants were omitted as outliers (as determined by values more than three standard deviation from the mean).

### Associations between cognitive impairment, HIV and CSVD status

**Table 2** shows the results of two-way ANOVA to measure the effects of HIV status, CSVD status, and their interactions on cognitive scores. Being HIV positive was associated with lower cognitive scores (p=0.0146, estimate: -2.7664, 95% CI: -4.9804 to -0.5525), and this effect was most prominent and statistically significant for the total z-scores and two sub-domains (Learning and Memory). CSVD status also trended toward a negative correlation with cognitive performance especially with Executive Function (**Supplementary Table 1**). Further it was worth noting that none of the interactions of HIV and CSVD status are significant, suggesting that the effects of these two factors to cognitive scores are mostly additive. **Supplementary Figure 1E** shows the total cognitive score when the cohort is categorized based on HIV and CSVD status into four groups. PWH with CSVD (-2.110 ± 4.126, n=77) had significantly reduced score as compared to PWoH without CSVD (1.781 ± 4.381, n=38, p=0.001) as well as PWoH with CSVD (1.115 ± 5.014, n=66, p=0.002). However, as shown in **Supplementary Figure 1A**, it should be noted that PWH with CSVD are a significantly older age group as compared to PWoH without CSVD and the effect of age on cognition may have significant impact on this comparison.

**Figure 1:**
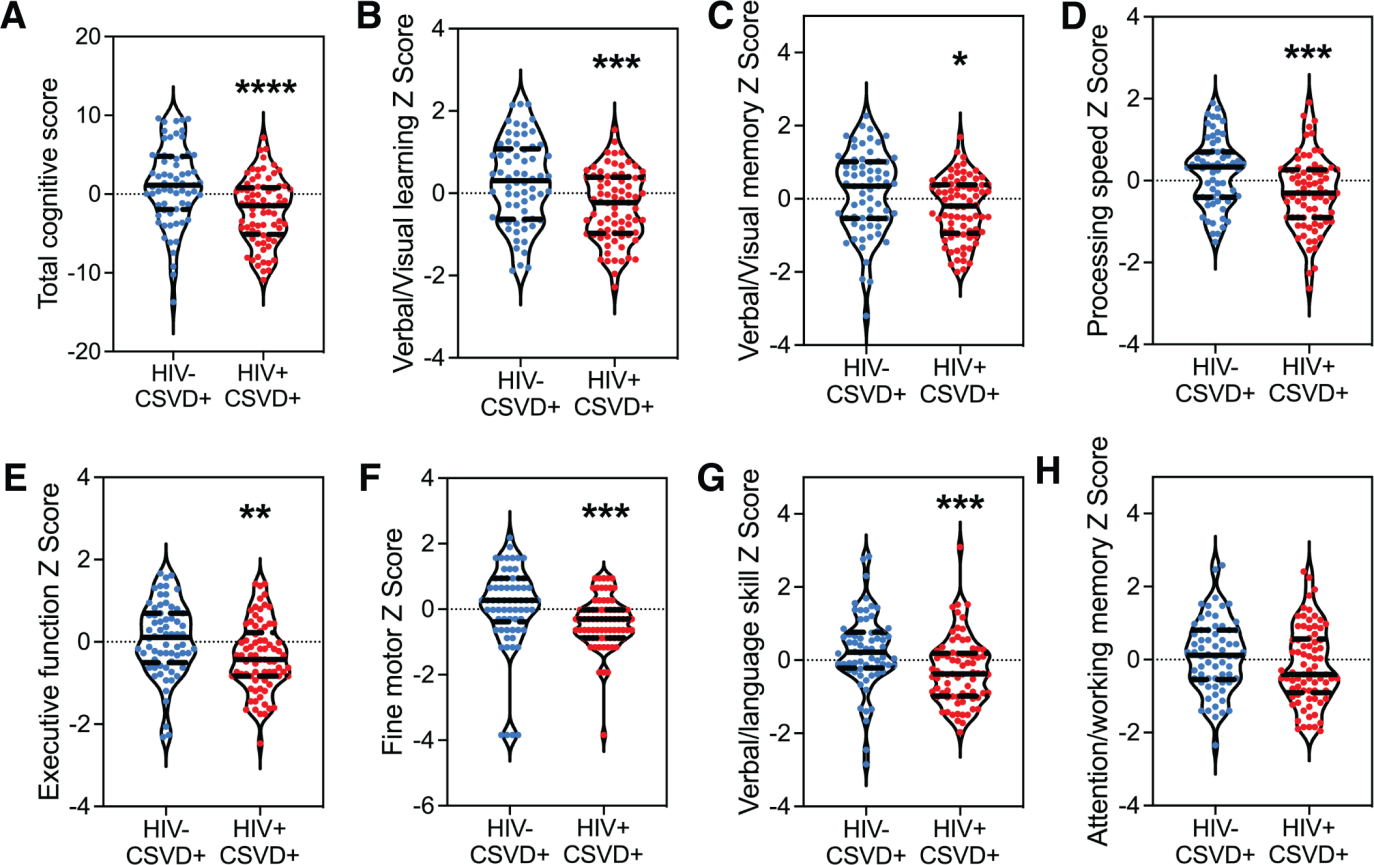
Neuropsychological battery domain scores in study participants. **(A)** Total cognitive score. Individual domain scores for **(B)** Verbal and Visual Learning, **(C)** Verbal and Visual Memory, **(D)** Processing speed, **(E)** Executive function, **(F)** Fine Motor Skill, **(G)** Verbal/language and, **(H)** Attention and working memory. Blue data points represent PWoH with CSVD group (n=66), and red represents PWH with CSVD group (n=77). Two group comparisons were made using Welch’s t-test. * p<0.05, ** p<0.01, *** p<0.001, **** p<0.0001.

The next set of analyses were performed only in participants with CSVD (PWoH with CSVD and PWH with CSVD). It is noteworthy that these two groups have similar age and RRS distribution (**Supplementary Figure 1**), indicating that the relative contribution of these covariates on CSVD status and cognitive impairment between both the groups is comparable. The results show that there was a significant decrease in total cognitive scores in PWH with CSVD group (-2.110 ± 4.126) as compared to PWoH with CSVD group (1.115 ± 5.014). Data is shown in **Figure 1A** for total cognitive score (p<0.0001, 95% CI for mean difference: -4.761 to -1.690). With the exception of attention/working memory domain, all the individual cognitive domains showed similar results (**Figures 1A–H**).

### Associations between blood biomarkers, HIV and CSVD status

**Figure 2:**
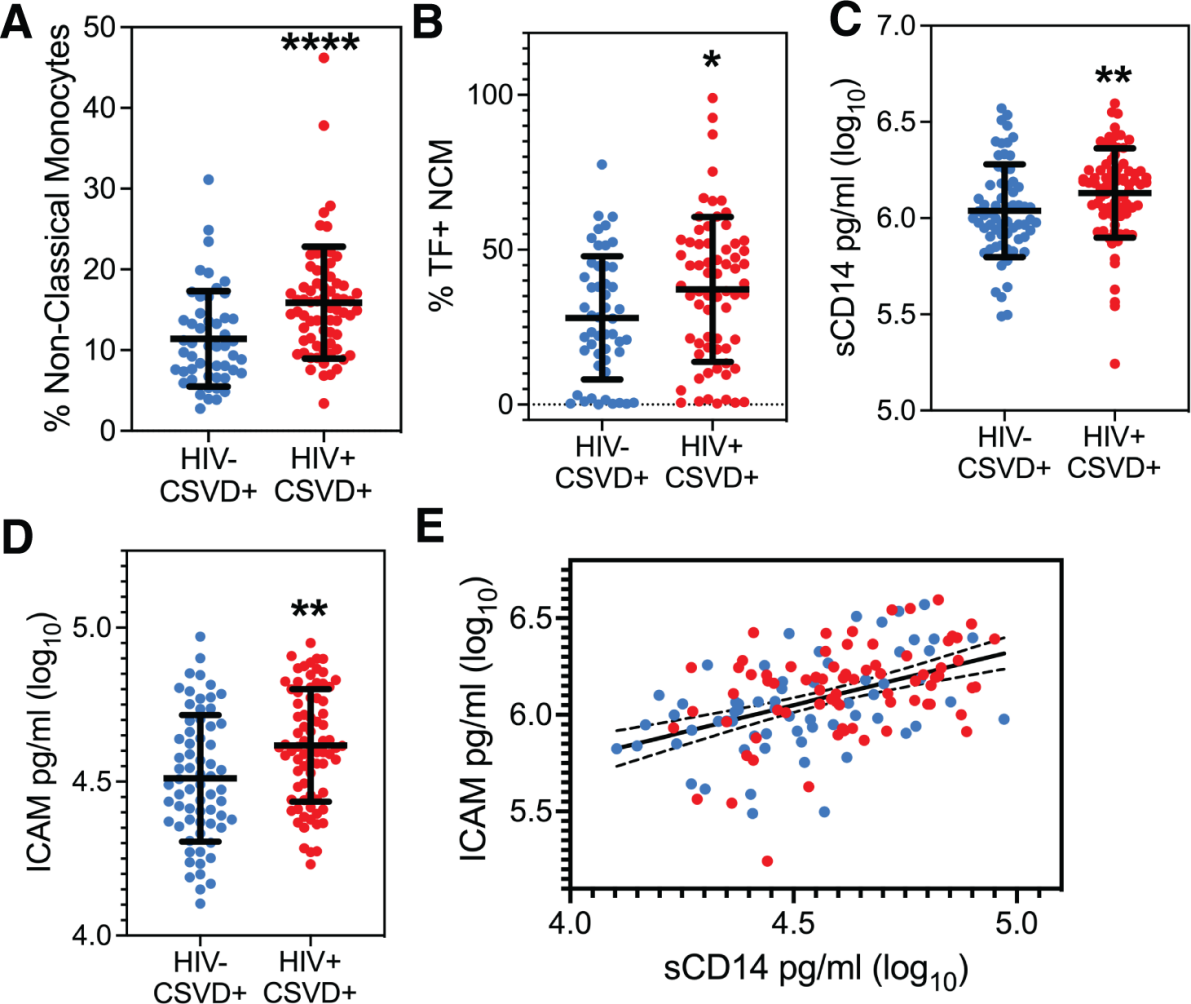
Peripheral monocyte levels and markers of monocyte and endothelial cell activation. **(A)** Circulating levels of NCM and, **(B)** Percentages of NCMs expressing TF were significantly higher in PWH with CSVD group as compared to PWoH with CSVD group. Plasma levels of **(C)** sCD14, and **(D)** ICAM were higher in the PWH WITH CSVD group as compared to the PWoH with CSVD group. **(E)** sCD14 and ICAM levels showed a robust positive correlation, indicating that monocyte and endothelial activation co-occur (p<0.0001, r_s_=0.4708). Blue data points represent the PWoH with CSVD group (n=66), and red represents the PWH with CSVD group (n=77). Two group comparisons were made using Mann Whitney U test. Correlation between sCD14 and ICAM was done via the Spearman test. * p<0.05, ** p<0.01, **** p<0.0001.

A two-way ANOVA of HIV status and CSVD and their interaction on monocyte phenotype showed that HIV status was positively associated with levels of NCMs (p=0.0473, estimate: 3.2130, 95% CI: 0.0387 to 6.3874) and there was no significant interaction between HIV status and CSVD status. **Supplementary Figure 1F** shows NCM levels when the cohort is categorized based on HIV and CSVD status into four groups. PWH with CSVD (14.13 ± 5.625, n=68) had significantly reduced score as compared to PWoH without CSVD (10.92 ± 4.207, n=31, p=0.0006) as well as PWoH with CSVD (11.4 ± 5.915 n=50, p=0.0012). Comparison between the two age matched CSVD+ groups exhibited that NCM levels were significantly higher in PWH with CSVD (n=68, median: 15.12 ± 6.929) as compared with PWoH without CSVD (n=50, median: 10.49 ± 5.915, p<0.0001, 95% CI of difference in median: 2.368 to 6.560, **Figure 2A**). No significant differences were observed in levels of intermediate (**Supplementary Figure 2A**) or classical monocyte subsets (**Supplementary Figure 2B**). Among the different monocyte activation markers measured, NCMs in PWH with CSVD group expressed higher levels of tissue factor (mean: 37.20 ± 23.42), as compared to PWoH with CSVD group (mean: 27.97 ± 19.88, **Figure 2B**, p=0.0233, 95% CI for mean difference: 1.276 to 17.17). No significant differences were observed in the expression of other monocyte activation markers in either NCM or other monocyte subsets.

Two-way ANOVA analysis for the entire cohort, using soluble markers of monocyte and endothelial activation, showed that HIV status was positively associated with sCD14 (p=0.0070, estimate:0.1577, 95% CI: 0.0436 to 0.2718) while CSVD status did not show any significant association. Results from two group comparisons (PWH with CSVD vs. PWoH with CSVD) showed that PWH with CSVD participants had significantly increased levels of sCD14 [median (log_10_ values): 6.175 ± 0.2321)] and ICAM levels [median (log_10_ values): 4.612 ± 0.1823)] than the PWoH with CSVD group (sCD14 median: 6.00 ± 0.2409, ICAM median: 4.490 ± 0.2056). Data is shown in **Figure 2C** for sCD14 (p=0.0033, 95% CI of median difference: 0.04 to 0.1877) and **Figure 2D** for ICAM (p=0.0015, 95% CI of median difference: 0.0420 to 0.1814). Comparisons for the two groups with Osteoprotegerin, CD163, LpPLA2 and VCAM showed no statistically significant differences (**Supplementary Figures 2C–F**).

Linear regression analysis for the entire cohort showed a significant association of sCD14 with ICAM, (p<0.0001, estimate: 0.5163, 95% CI: 0.3550 to 0.6777) and with HIV status (p=0.0453, estimate:0.0632, 95% CI: 0.0013 to 0.1251). This result was further confirmed in participants with CSVD. We found that Spearman correlation between sCD14 and ICAM levels was significant (p<0.0001, r_s_=0.4708, 95% CI: 0.3263 to 0.5938, **Figure 2E**).

### Circulating levels of non-classical monocytes might be predictive of cognitive performance in HIV-associated CSVD

**Figure 3:**
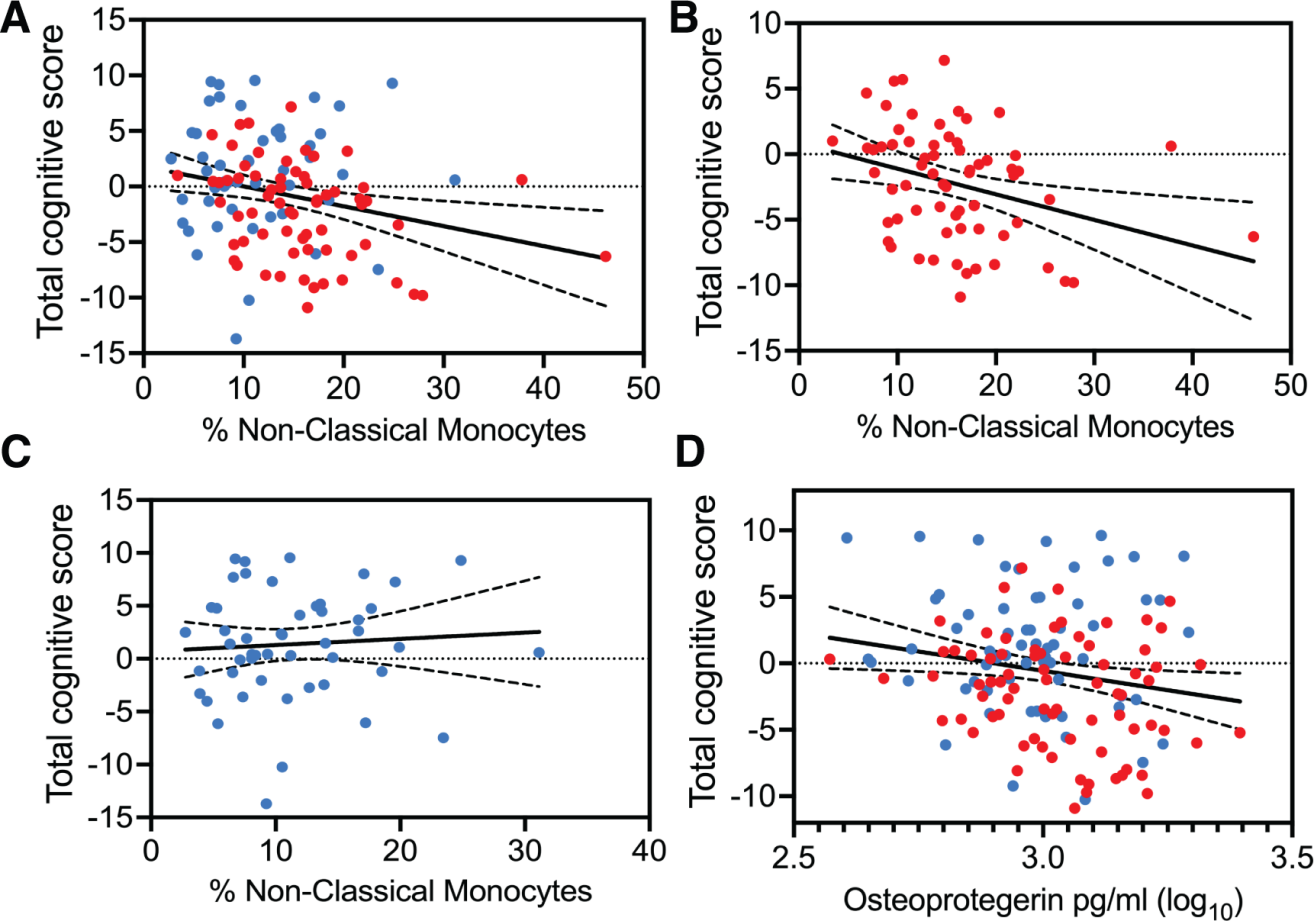
Correlation analysis between total cognitive Z score and blood markers of monocyte and endothelial cells. Percentages of NCMs and total cognitive score showed statistically significant negative correlation **(A)** when data from the CSVD+ groups was pooled together (p=0.0043, r_s_ = -0.2631). **(B)** This correlation became stronger among PWH with CSVD (p=0.0032, r_s_ = -0.3579). **(C)** There was no correlation when data from only PWoH with CSVD individuals was used (r_s_ = 0.09705). **(D)** Osteoprotegerin showed a mild negative correlation with total cognitive scores only when data from both CSVD+ groups was pooled together (p=0.0409, r_s_=-0.1743). Blue data points represent the PWoH with CSVD group (n=66), and red represents the PWH with CSVD group (n=77). Correlations were done via the Spearman test.

All three subsets of monocytes and as well as soluble markers of endothelial and monocyte activation were assessed against cognitive scores (total and individual domain scores) using multiple regression analysis. Results showed that HIV status had the strongest association with total cognitive performance, followed by osteoprotegerin and NCMs (**Table 3**). Two group comparisons within CSVD+ groups showed that NCM levels and total cognitive score were inversely correlated with each other (**Figure 3A**, p=0.0043, r_s_ = -0.2631, 95% CI: -0.4295 to -0.0794). This correlation was even stronger in PWH with CSVD group (**Figure 3B**, p=0.0032, r_s_ = -0.3579, 95% CI: -0.5572 to -0.1197), while there was no significant correlation in PWoH with CSVD group (**Figure 3C**, p=0.5025, r_s_ = 0.09705, 95% CI: -0.1945 to 0.3728). **Table 4** shows significant correlation coefficients for NCMs and individual cognitive domains in combined CSVD+ groups and PWH with CSVD group alone. The correlations are stronger in most domains for the PWH with CSVD group compared to the combined CSVD groups.

**Table 4:** Spearman correlation of Individual cognitive domain scores as a function of NCM levels.

Cognitive domain	Both CSVD+ groups			PWH WITH CSVD only		
	r_s_	p-value	95% CI	r_s_	p-value	95% CI
Processing speed	-0.2453	0.0080	-0.4138 to -0.06046	-0.3216	0.0085	-0.5282 to -0.07907
Executive Function	-0.2493	0.0070	-0.4174 to -0.06478	-0.3684	0.0023	-0.5655 to -0.1316
Fine motor Skill	-0.1201	0.0068	-0.4181 to -0.06564	-0.2138	0.0848	-0.4393 to 0.03708
Verbal/language	-0.2125	0.0220	-0.3847 to -0.02590	-0.3049	0.0128	-0.5147 to -0.06056

Among other markers, osteoprotegerin showed mild negative correlation with total cognitive score only when data from PWoH with CSVD and PWH with CSVD groups were pooled together (**Figure 3D**, p=0.0409, r_s_=-0.1743, 95% CI: -0.3361 to -0.0023). Further, we correlated the blood markers and the total cognitive score with absolute WMH volumes, however, no significant correlations between these measures were found (data not shown). It should be noted that Fazekas scores for most of the participants with CSVD were between 1 and 2 indicating that the volume of WMH was relatively small.

Lastly, we conducted two group analysis to investigate the effect of virus associated covariates, such as CD4 T cell counts, viral loads and protease inhibitor usage on levels of NCM as well as total cognitive score, since these factors have been known to be associated with inflammation and cognition. Results indicated that there was no significant difference in the levels of NCM or the total cognitive score when PWH were categorized into two groups either based on detectable vs. undetectable viral load (**Supplementary Figure 3A**, p= 0.4246, **Supplementary Figure 3B**, p=0.5447 respectively) or CD4 T cell counts below 500 cells/mm^3^ or equal to/more than 500 cells/mm^3^ (**Supplementary Figure 3C**, p= 0.5626, **Supplementary Figure 3D**, p= 0.83 respectively) or PWH with or without protease inhibitor treatment (**Supplementary Figure 3E**, p= 0.5737, **Supplementary Figure 3F**, p= 0.6764). These results suggest that the contribution of these variables on NCM levels and cognition may be minimal in our cohort.

## Discussion

Our results indicate that among the populations of monocytes, NCMs represent a common denominator linking inflammation, CSVD, and cognitive impairment in PWH. NCMs, alternatively called patrolling monocytes, are responsible for the resolution of inflammation during homeostasis (Auffray et al., 2007). However, multiple studies have also shown that NCMs are equally capable of partaking in the pathogenesis of inflammatory diseases, including vascular disease [reviewed in (Narasimhan et al., 2019)].

With regards to HIV infection, monocytes expressing CD16 have been considered to be responsible for seeding and maintenance of CNS viral reservoir as well as for increased neuroinflammation during HIV infection (Koenig et al., 1986; Fischer-Smith et al., 2001; Burdo et al., 2010; Valcour et al., 2012; Veenstra et al., 2017). Veenhuis et al. reported that 40-50% of PWH exhibited intact viral genome in monocytes and this virus could be reactivated using macrophages derived from such monocytes (Veenhuis et al., 2023). A study by Shikuma et al. showed that higher antiretroviral monocyte efficacy score was linked with better cognitive performance (Shikuma et al., 2012). HIV DNA levels in monocytes and soluble CD14 were found to be associated with NCI in PWH (Kusao et al., 2012; Munoz-Nevarez et al., 2020). With respect to vascular disease, increased circulating NCM and IMC levels have been shown to be associated with severity of coronary artery disease (Ozaki et al., 2012) or predict the progression of carotid artery bifurcation intima media thickness (Chow et al., 2016). While in another study only IMCs were shown to be an independent predictor of cardiovascular events (Rogacev et al., 2012). Several reports have shown either higher levels of circulating monocytes or soluble monocyte products to be elevated during cognitive decline associated with sepsis, neuropathic injury, Alzheimer’s disease, and stress (Weber et al., 2017; Andonegui et al., 2018; Berger et al., 2019; Kuan et al., 2021; Mai et al., 2021; Munawara et al., 2021).

Our finding in PWH differ from those of Noz et al (Noz et al., 2018), where they showed in a much smaller sample size that IMCs and not NCMs were associated with CSVD. Our results also differ from Veenhuis et al., who showed that higher IMC levels were associated with lower global neuropsychological function score in two small cohorts of 25 and 18 women, respectively (Veenhuis et al., 2021). These reports highlight the need for a better characterization of monocyte subsets. Since monocytes sequentially mature from classical to IMCs to NCM phenotypes, using only CD14 and CD16 to define the three subsets can introduce a study-to-study variation in how the subsets are demarcated. Inclusion of additional markers such as CD11c, CD36, CCR2, and CD163 in flow cytometry panels and/or using single cell RNAseq analysis might be needed to resolve the heterogeneity within monocyte subsets (Hamers et al., 2019). Nevertheless, all of these studies, including ours, underscore that monocytes which express CD16 (IMCs and NCMs) are associated with vascular disease.

In addition to increased levels of NCMs among PWH, we show that NCMs also expressed higher levels of tissue factor (TF), which is the primary activator of intrinsic and extrinsic coagulation pathways (Camerer et al., 1996; Bode and Mackman, 2014). In the context of CSVD, increased TF+ NCM could contribute to strokes (Iacoviello et al., 2015) in PWH.

Another interesting insight into the interplay between peripheral and neuronal inflammation was provided by the assessment of soluble markers of monocyte and endothelial activation, ICAM and sCD14, which were found to be increased in PWH with CSVD. Both of these markers positively correlated with each other very strongly, indicating that monocyte activation coincided with endothelial activation. The interaction between activated endothelial cells and monocytes is likely to contribute to the transmigration of NCMs, and subsequent neuroinflammation. Of interest, osteoprotegerin, an endothelial marker associated with CSVD (Shoamanesh et al., 2015), was associated with cognitive performance independently of HIV and CSVD status. These markers have been previously linked with CSVD in general population (Rouhl et al., 2012; Shoamanesh et al., 2015) and also with development and progression of HIV-associated atherosclerosis (Ross et al., 2010; Montagnana et al., 2013; Ronsholt et al., 2013; D’Abramo et al., 2014) as well as cognitive impairment (Saloner et al., 2022; Guha et al., 2023).

In our study, cognitive performance was strongly associated with HIV status rather than the presence of CSVD but with one exception, executive function. This finding is consistent with previous reports of vascular cognitive decline in CSVD. A study by Uiterwijk reported that in individuals with hypertension, CSVD MRI measures associated with Cognitive impairment in the executive domain (Uiterwijk et al., 2016). Similarly, Barucci et al. reported that hypertension related microangiopathy correlated with mainly attentional/executive cognitive deficits while cerebral amyloid angiopathy was associated with semantic memory deficits (Barucci et al., 2024). With respect to HIV, postmortem studies have showed that mild CSVD was present in 24.8% and moderate/severe CSVD in 47.4% of the cases examined (Soontornniyomkij et al., 2014). A study from the Hawaii Aging with HIV Cohort Study (HAHCS) found white matter hyperintensity in 48% of the subjects that had neuroimaging (McMurtray et al., 2008). Plenty of other studies including a recent one from Alakkas et al. have documented CNS injury and specifically white matter microstructure changes in PWH (Filippi et al., 2001; Wu et al., 2006; Chang et al., 2008; Pfefferbaum et al., 2009; Tate et al., 2010; Zhu et al., 2013; Alakkas et al., 2019). Sanford et al. reported that HIV and CSVD had independent but additive contributions to cognitive impairment and did not show significant interaction (Sanford et al., 2019). In our study deficits in other cognitive domains seem to be less impacted by CSVD. However, this may reflect the overall mild CSVD (primarily a Fazekas score of 1 and 2). It is likely that with worsening of the CSVD burden multiple cognitive domains will be affected (Vasquez and Zakzanis, 2015; Hamilton et al., 2021; Yang et al., 2023). Interestingly, a recent study on an aging population did not find any significant association between white matter hyperintensity volume and cognitive function or cognitive decline (Wang et al., 2024). It is possible that other feature of CSVD, such as brain atrophy, may be necessary to lead to cognitive impairment.

There are some limitations to our study. While PWH were well matched to PWoH for age, sex, and Reynold’s cardiovascular risk score, a larger sample size and a more equal distribution of sex-at-birth would have been preferable. Unfortunately, the HIV population in the recruitment geographic area is heavily skewed toward males. Every effort was made to enroll female study participants, which led to at least 25% female study participants in each cohort. Similarly, the numbers of participants to who reported to be of African American origin and/or Hispanic were very limited and thus we could not include race/ethnicity as a covariate in statistical analysis. Of notice, the study excluded participants with uncontrolled cardiovascular risk factors thus potentially limiting the generalizability of the results. At the same time, PWH cohort had more incidence of diabetes and Hepatitis C infection as compared to PWoH, however we did not have sufficient power to statistically investigate the impact of these comorbidities on study outcomes. An additional limitation is in the collection of blood samples when phenotyping monocytes. As it is often done, blood collection is performed once per visit. However, given the course of the median lifespan of monocytes and the maturation process from classical monocytes to non-classical monocytes, more dynamic measurements (2-3 times over 6-7 days) may better explain changes in chronic inflammation as it occurs in PWH. Neurofilament light chain (NFL)- a marker of neuronal injury, Glial Fibrillary Acidic Protein (GFAP)- a marker of glial injury, and phosphorylated tau-a marker of synaptic loss have been reported to be increased in PWH in plasma and CSF, especially in cART-naïve individuals (Peterson et al., 2014; Gisslen et al., 2016; Guha et al., 2019; Ozturk et al., 2019; Alagaratnam et al., 2022; de Menezes et al., 2022; Ellis et al., 2022; Sathler et al., 2022; Ripamonti et al., 2023). However, these reports have not been consistent in the findings, and we did not evaluate these markers in this study since our focus was on monocyte and endothelial activation.

In conclusion, elevated levels of NCMs in PWH may represent a common pathway to neuroinflammation and CSVD leading to cognitive impairment. The pending longitudinal analyses may further inform on the association of NCM and cognition. However, the low to moderate correlation of NCM and cognition suggests that other co-contributors may be at play, for example, neutrophils and T cells. Additional investigations with larger cohorts are warranted to further confirm the findings of this study. In PWH with mild CSVD, cognitive dysfunction is driven by HIV infection with a smaller contribution of CSVD. However, with aging, the burden of CSVD will continue to increase, as will the contribution of CSVD to cognitive impairment in PWH. There are currently no therapies that directly target monocyte populations. Instead, there are therapies that address different aspects of inflammation such as statins, aspirin, celecoxib etc. Clinical management of CSVD is also limited to treatment of traditional risk factors such as diabetes, hypertension, hyperlipidemia. In this regard, our results indicate that peripheral levels of NCMs may serve as a useful biomarker to assess response to intended CSVD treatments or to monitor the progression of CSVD. Moreover, in depth transcriptomic and proteomic characterization of monocyte subpopulations and their association with HIV-associated vascular disease may generate information about critical signaling pathways in monocytes that could be targeted to generate therapeutic strategies to alleviate these co-morbidities.

## Data Availability

The original contributions presented in the study are included in the article/**Supplementary Material**. Further inquiries can be directed to the corresponding authors.
