# Supplementary material for: Non-classical monocyte levels correlate negatively with HIV-associated cerebral small vessel disease and cognitive performance
Source: Front Cell Infect Microbiol. 2024 Oct 23;14:1405431. doi: 10.3389/fcimb.2024.1405431 (PMC11537857; doi:10.3389/fcimb.2024.1405431)
Supplement: Supplementary file 1 [file DataSheet1.pdf]

Supplementary Figure 1: Age, Reynold's risk score (RRS), and white matter hyperintensity (WMH) lesion burden in study participants. **A.** Age distributions of study participants indicate that both groups with CSVD are matched by age. **B.** RRS distribution of study participants indicates that both groups with CSVD have similar cardiovascular risk burden. **C.** PWH have a higher volume of WMH lesions as compared to HIV- groups, irrespective of CSVD status. **D.** PWH with CSVD have higher levels of WMH burden as compared to the HIV-CSVD+ group. **E**. PWH with CSVD exhibit significantly decreased total cognitive score as compared to PWoH with and without CSVD. Light blue data points represent the HIV-CSVD- group (n=38), dark blue represents the HIV-CSVD+ group (n=66), orange represents the HIV+CSVD- group (n=28), and red represents the HIV+CSVD+ group (n=77). Four group comparisons were made using two-way ANOVA followed by Tukey's multiple comparisons test. Two group comparisons by Welch's t-test.

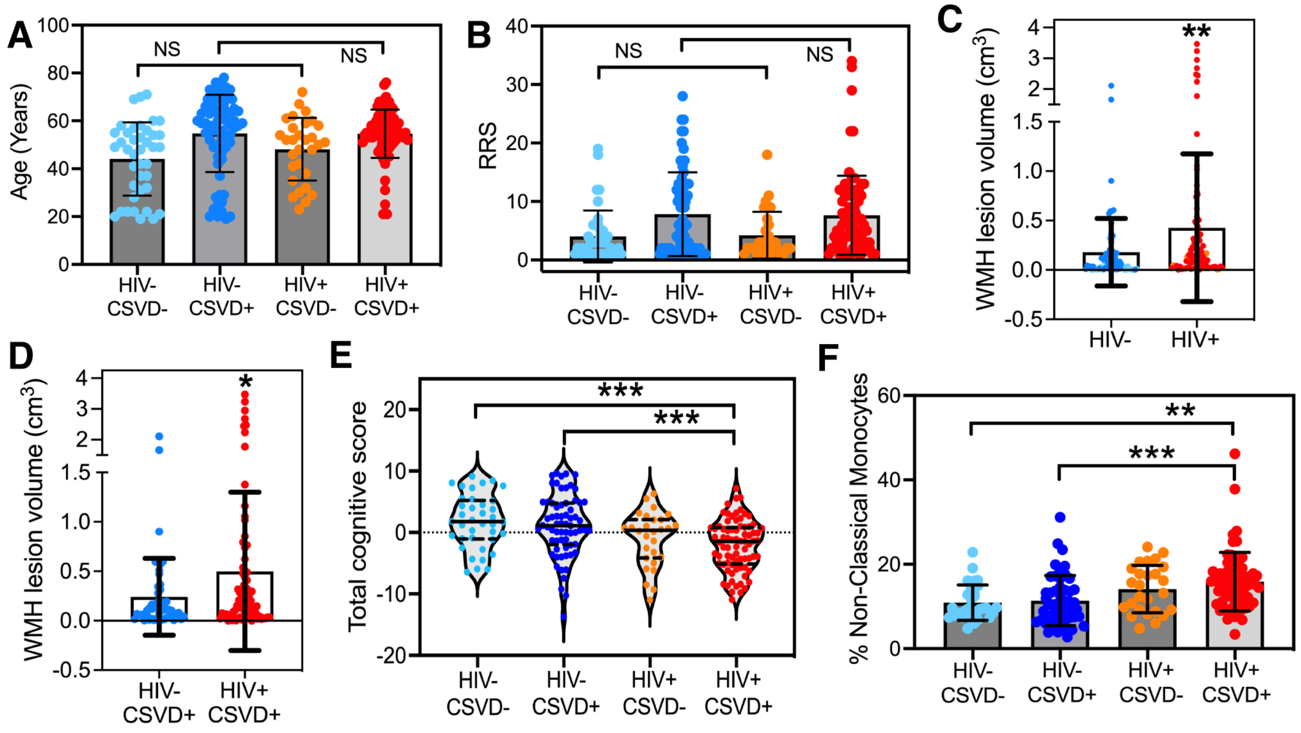

Supplementary Figure 2: Monocyte and endothelial markers. Levels of **A.** intermediate and **B.** classical monocytes were not different between HIV+CSVD+ and HIV-CSVD+ groups. Plasma levels of **C**. Osteoprotegerin, **D.** CD163, **E.** LpPLA2, and **F.** VCAM were not statistically different between the two groups. Blue data points represent the HIV-CSVD+ group (n=66), and red represents the HIV+CSVD+ group (n=77). Two group comparisons were made by the Mann Whitney test.

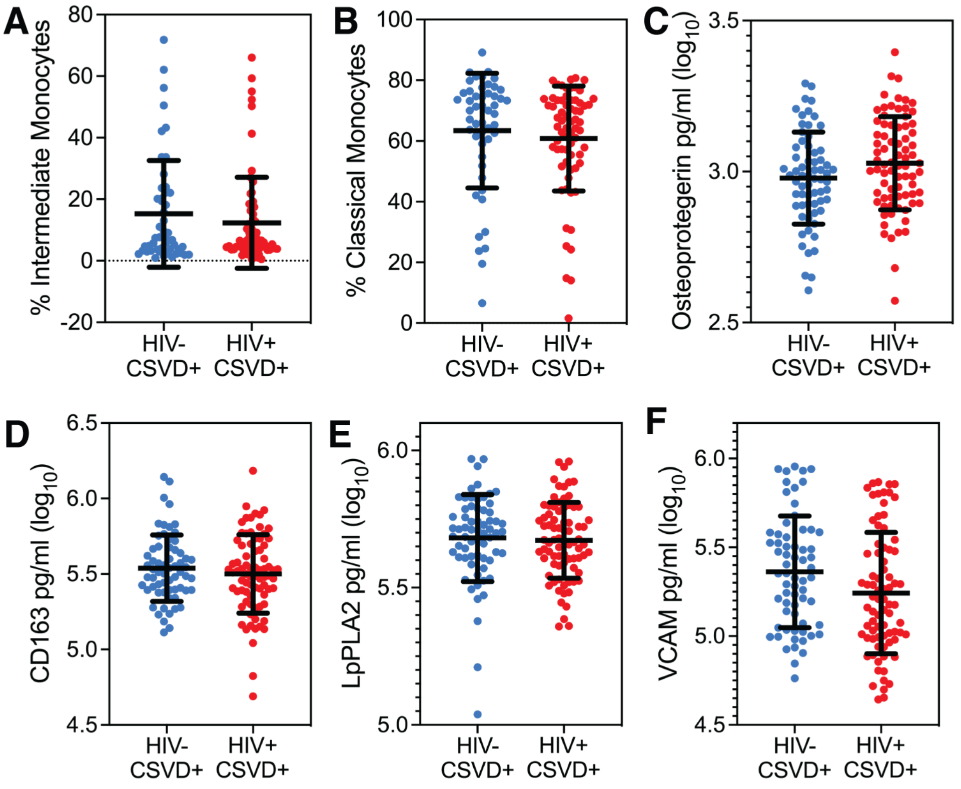

Supplementary Figure 3: NCM and Total Z score comparisons by viral load, CD4 counts and PI usage. A. Levels of NCM and B. total cognitive score comparison between participants with undetectable viral load (VL<20) and detectable viral load. C. Levels of NCM and D. total cognitive score comparison between participants with CD4 T cell counts below and ≥ 500 cells/mm^3. E. Levels of NCM and F. total cognitive score comparison between participants on protease inhibitors (PI) and those not on protease inhibitors were not significantly different. Orange data points represent HIV+CSVD- group and red data points represent HIV+CSVD+ group. Two group comparisons were made by Mann Whitney U test.

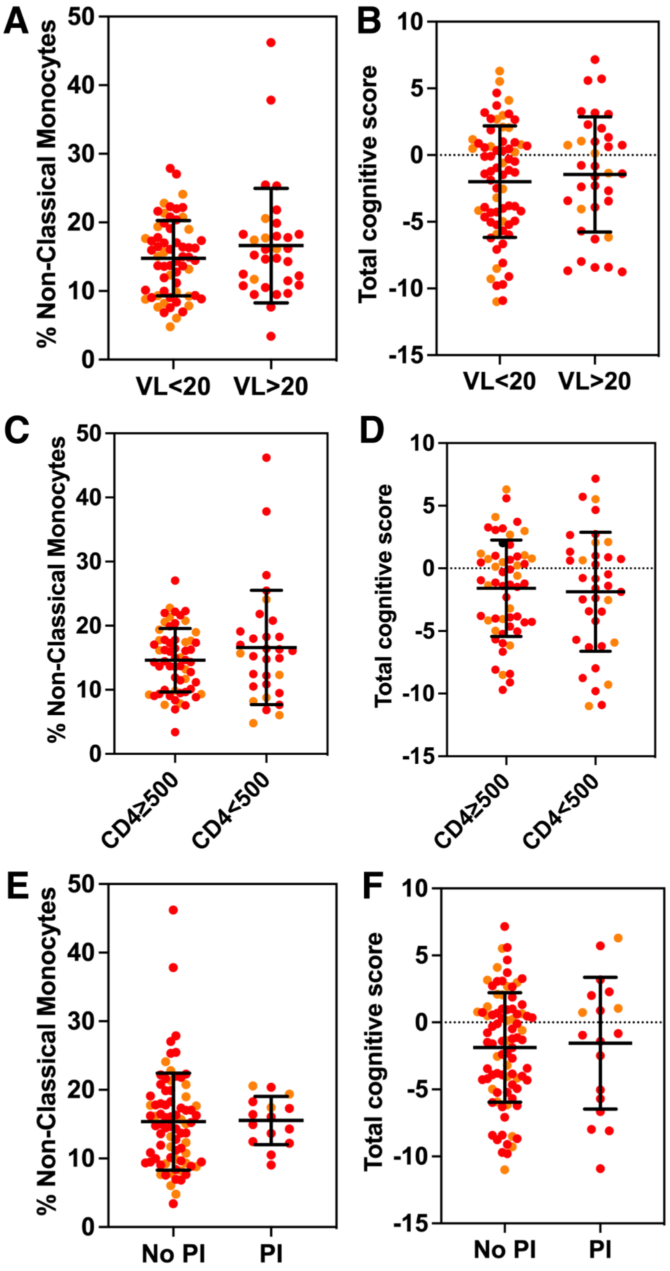

Supplementary Table 1: Two-way ANOVA to measure the effects of HIV status, CSVD status, and their interactions on individual cognitive domain scores.

Domain Name		Estimate	Std. Error	t value	P value	BH Adjusted P value	2.50%	97.50%
Verbal/visual learning	HIV Status	**-0.6777**	**0.2285**	**-2.9657**	**0.0034**	**0.0236**	**-1.1282**	**-0.2272**
	CSVD Status	0.023	0.1868	0.1234	0.9019	0.9019	-0.3453	0.3914
	HIV-CSVD Interaction	0.0982	0.2755	0.3564	0.7219	0.9304	-0.445	0.6413
Verbal/visual memory	HIV Status	**-0.5146**	**0.2388**	**-2.155**	**0.0323**	0.1132	**-0.9854**	**-0.0438**
	CSVD Status	-0.0527	0.1952	-0.2699	0.7875	0.9019	-0.4377	0.3322
	HIV-CSVD Interaction	0.0768	0.2879	0.2666	0.79	0.9304	-0.4909	0.6444
Processing speed	HIV Status	-0.4048	0.2332	-1.736	0.0841	0.1614	-0.8645	0.0549
	CSVD Status	-0.1138	0.1907	-0.5968	0.5513	0.7719	-0.4897	0.2621
	HIV-CSVD Interaction	-0.1041	0.2811	-0.3703	0.7115	0.9304	-0.6584	0.4502
Executive function	HIV Status	-0.3542	0.2258	-1.5686	0.1183	0.1656	-0.7994	0.091
	CSVD Status	-0.3552	0.1846	-1.9235	0.0558	0.3906	-0.7192	0.0089
	HIV-CSVD Interaction	-0.07	0.2723	-0.257	0.7975	0.9304	-0.6068	0.4668
Fine motor skill	HIV Status	-0.1358	0.2455	-0.553	0.5809	0.5809	-0.6198	0.3483
	CSVD Status	-0.1931	0.2007	-0.962	0.3372	0.7719	-0.5889	0.2027
	HIV-CSVD Interaction	-0.3094	0.296	-1.0453	0.2971	0.9304	-0.893	0.2742
Verbal/language skill	HIV Status	-0.4036	0.2386	-1.6916	0.0922	0.1614	-0.8741	0.0668
	CSVD Status	0.1705	0.1951	0.8742	0.383	0.7719	-0.2141	0.5552
	HIV-CSVD Interaction	-0.1459	0.2877	-0.5071	0.6127	0.9304	-0.7131	0.4213
Attention/working memory	HIV Status	-0.2758	0.25	-1.1032	0.2713	0.3165	-0.7687	0.2171
	CSVD Status	-0.1447	0.2044	-0.7078	0.4799	0.7719	-0.5477	0.2583
	HIV-CSVD Interaction	-0.0043	0.3014	-0.0142	0.9887	0.9887	-0.5985	0.59
